# Preoperative malnutrition is a risk factor for prolonged postoperative ileus for patients undergoing gastrointestinal surgery

**DOI:** 10.3389/fnut.2025.1561264

**Published:** 2025-04-09

**Authors:** Zhenming Zhu, Baoguo He, Juan He, Xuan Ma, Qun Gao, Yinghui Huang, Yuning Chu, Li Ma

**Affiliations:** ^1^Department of Gastroenterology, The Affiliated Hospital of Qingdao University, Qingdao, China; ^2^Department of Clinical Nutrition, The Affiliated Hospital of Qingdao University, Qingdao, China

**Keywords:** preoperative malnutrition, prolonged postoperative ileus, gastrointestinal surgery, PG-SGA, complications

## Abstract

**Background:**

Prolonged postoperative ileus (PPOI), a common complication after gastrointestinal (GI) surgery, seriously affects the postoperative recovery rate. However, there are few previous studies on the effect of preoperative nutritional status on the occurrence of PPOI in patients with GI cancer.

**Objective:**

To evaluate the value of preoperative nutritional status for predicting the occurrence of PPOI in patients undergoing GI surgery.

**Methods:**

We retrospectively analyzed the clinical data of GI cancer patients who were admitted to our hospital between June 2021 and June 2023. The nutritional status of all patients was assessed using the Nutritional Risk Screening 2002 (NRS2002) and the Patient-Generated Subjective Global Assessment (PG-SGA). The independent risk factors for PPOI identified via univariate and multivariate logistic regression analyses were used to establish nomogram for the prediction of PPOI.

**Results:**

The clinical data of 310 patients with GI cancer who underwent surgical resection were analyzed. PG-SGA score, serum albumin concentration, hemoglobin concentration, operation time, tumor stage, and previous abdominal surgery are independent risk factors for PPOI. The nomogram developed to predict PPOI performed well [area under the curve (AUC) = 0.835]. The calibration curve showed high consistency between the observed and predicted results. The decision curve analysis (DCA) revealed that the nomogram was clinically useful. The predictive ability of this nomogram is better than that of albumin level and PG-SGA score.

**Conclusion:**

The preoperative nutritional status of GI cancer patients has a significant effect on the occurrence of PPOI. The nomogram developed in this study accurately predicted PPOI in GI surgery patients.

## Introduction

Postoperative ileus (POI) is the temporary inhibition of gastrointestinal (GI) motility commonly caused by nonmechanical factors after GI surgery (1). POI usually does not last more than 3 days; however, if it lasts longer than 3 days, it is considered prolonged postoperative ileus (PPOI) (2). PPOI is characterized by GI symptoms such as abdominal distension, nausea, vomiting, oral intake intolerance, and delayed exhaust and defecation (3). PPOI seriously affects the speed of postoperative recovery, prolongs the length of hospital stay, increases medical costs, and imposes a large burden on patients as well as society and medical systems (4). At present, there is still a lack of effective treatment options for PPOI. Therefore, it is very important to find the risk factors for PPOI and identify high-risk patients for PPOI, so as to prevent the occurrence of PPOI through early intervention (5).

Gastric cancer (GC) and colorectal cancer (CRC) are two of the most common malignancies in the world, accounting for 5.6 and 10.0% of all malignancies, respectively, and are also two of the most common causes of cancer-related death (6). As the most common GI cancers, GC and CRC increase the risk of malnutrition (7). At present, the nutritional assessment of cancer patients generally involves anthropometric measurements including serum biochemical indicators, nutritional risk screening 2002 (NRS 2002) and patient-generated subjective global assessment (PG-SGA) (8). According to the expert consensus of the European Society for Parenteral and Enteral Nutrition (ESPEN), NRS 2002 is the first choice for assessing nutritional status, and the PG-SGA is the first choice for nutritional assessment in patients with malignant tumors (9).

Previous studies have shown that preoperative malnutrition increases the relative risk of postoperative complications (10). Kang et al. reported that preoperative hypoalbuminemia increased the risks of overall and major postoperative complications and that hypoalbuminemia was an independent predictor of overall and major complications (11). Liang et al. reported that the preoperative albumin level was an independent predictor of PPOI in patients who underwent GI surgery (12).

However, to date, there have been no studies on the relationships between the NRS-2002 score, PG-SGA score and PPOI in patients with GI cancer. In addition, there are few studies on the indicators of nutritional status other than serum albumin concentration for the prediction of PPOI. Therefore, the aim of this study was to evaluate the value of the NRS-2002 score, PG-SGA score, and nutritional indicators for predicting PPOI in GC and CRC patients undergoing radical surgery and, at the same time, to develop relevant predictive models and determine their predictive power.

## Materials and methods

### Patients

The clinical data of patients who were diagnosed with GC or CRC at the Affiliated Hospital of Qingdao University between June 2021 and June 2023 were retrospectively analyzed. The inclusion criteria were as follows: (1) patients with a pathological diagnosis of colorectal adenocarcinoma or gastric adenocarcinoma; (2) patients with completely resectable tumors; and (3) patients who signed informed consent forms. The exclusion criteria were as follows: (1) patients who underwent emergency surgery; (2) patients who required laparoscopic conversion to open surgery (Patients had to be converted to open surgery due to the difficulty of complete resection of the tumor by laparoscopic surgery or intraoperative bleeding); (3) patients who underwent multiple organ resection; (4) patients with a postoperative intra-abdominal infection or intra-abdominal hemorrhage; and (5) patients who were diagnosed with other diseases that can easily cause GI motility insufficiency, such as diabetes mellitus, severe cardiopulmonary disease, renal insufficiency, and thyroid disease. This study was approved by the Ethics Committee of the Affiliated Hospital of Qingdao University, and the informed consent was waived by the Institutional Review Board of the Affiliated Hospital of Qingdao University.

### Definition of PPOI

PPOI was defined on the basis of a systematic review and global survey (13). Patients were diagnosed with PPOI if they met two or more of the following five criteria on postoperative day 4 or after: (1) nausea or vomiting lasting 12 h or more without relief; (2) intolerance to solid or semisolid oral diets; (3) persistent abdominal distention; (4) absence of stool and exhaust for 24 h or more; and (5) intestinal obstruction on plain abdominal radiography or computed tomography (CT) scan. We adopted this definition, and two investigators independently diagnosed PPOI.

### Assessment method

All patients completed the NRS2002 after admission. The NRS 2002 consists of three parts: nutritional status assessment (weight loss, body mass index [BMI] and food intake), disease severity (impact of the disease on stress level and metabolism) and age (age 70 years or older) (14). The score ranges from 0 to 7 points. Patients with a score ≥ 3 were considered at risk of malnutrition, whereas those with a score < 3 were considered at no risk of malnutrition. Assessments were performed blindly by two trained nurses followed by an audit by a clinical dietitian. The nurse had been doing this job for more than 1 year.

The PG-SGA is completed by the patients and the medical staff and consists of seven sections. The patient self-assessment includes weight changes, dietary intake, self-reported symptoms, activities and function, and the medical staff assessment includes nutrition-related disease status, metabolic status, and physical examination. The scores of the seven aspects range from 0 to 4 points, and each of the sums of the scores is divided into quantitative and qualitative evaluations, which are then used to guide the selection of the most appropriate interventions (15). PG-SGA scores of 0–1 indicated no need for nutritional support; scores of 2 to 3 indicated mild or suspected malnutrition; scores of 4 to 8 indicated moderate malnutrition; and scores above 9 indicated severe malnutrition (8, 16). PG-SGA assessments were performed by two registered clinical nutritionists trained in PG-SGA to ensure assessment accuracy. Patients underwent the assessment unaware of the specific content of this study to ensure the authenticity of the patient’s condition.

### Data collection

We collected basic data from patients with a diagnosis of GC or CRC, including sex, age, smoking habits, alcohol use, and previous abdominal surgery. Body mass index (BMI) was calculated from height and weight. According to the surgical records and pathological reports of the patients, the operation time, operation method (open or laparoscopic), and tumor type were collected, and tumor staging was performed according to the 7th edition of the International Union against Cancer tumor-node-metastasis classification of malignant tumors system (17). The NRS 2002 score, PG-SGA score and serum biochemical indicators such as total protein, albumin, prealbumin, triglyceride, total cholesterol, cholinesterase, uric acid, potassium (K^+^), magnesium (Mg^+^), phosphorus (P^+^), hemoglobin, and lymphocyte count were collected on admission. Serum biochemical parameters were measured in the fasting state of the patients.

### Establishment and validation of the nomogram

All variables were subjected to a univariable analysis, those with a *p* value <0.05 were incorporated into the multivariable logistic analysis to determine the independent risk factors affecting the occurrence of PPOI, and the independent risk factors were used to develop the nomogram. The area under the curve (AUC) of the receiver operating characteristic (ROC) curve was used to evaluate the predictive ability of the nomogram. The bootstrap method (1,000 bootstrap resamples) was used to conduct internal verification of the nomogram model. Decision curve analysis (DCA) was also performed to determine the clinical utility of the predictive model.

### Statistical analyses

Continuous variables are expressed as means ± SDs, and categorical variables are expressed as numbers and percentages. Independent t tests or Mann–Whitney U tests were used to compare continuous variables, whereas chi–square tests or Fisher’s exact tests were used to compare categorical variables. Multivariable logistic regression models were used to determine the odds ratio (OR), 95% confidence interval (95% CI), and P for each associated factor. A *p* value < 0.05 for two-sided tests indicated statistical significance. All analyses were performed with SPSS software (version 25.0, IBM Corp., Armonk, NY, United States), and R software (version 4.2.0, R Foundation, Vienna, Austria) was used for comprehensive statistical analysis of the collected data.

## Results

### Patient characteristics

**Figure 1:**
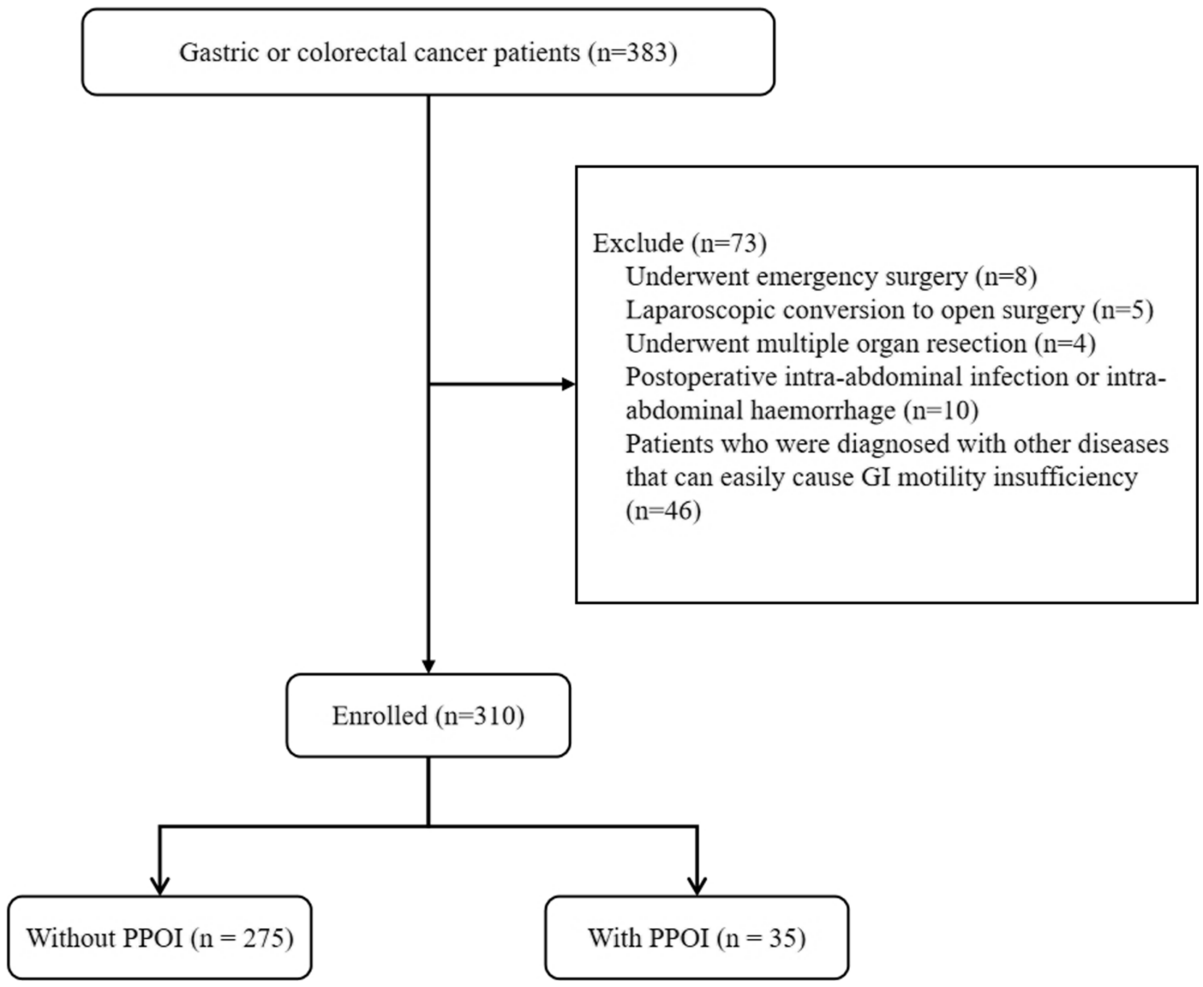
Diagram of patient enrollment in the study.

A total of 310 patients were enrolled in this study (Figure 1). The mean age of the enrolled patients was 61.74 ± 10.49 years, and most of the patients were male (64.8%). The proportions of patients with GC (50.6%) and CRC (49.4%) were similar. Fifty-seven (18.4%) patients had previously undergone abdominal surgery. The mean operation time was 190.06 ± 64.50 min. The overall PPOI rate was 11.3%. According to the NRS 2002, 54.2% of the patients were at risk of malnutrition, and 46.1% of the patients were not at risk of malnutrition. The PG-SGA evaluation revealed that 28.4% of patients scored 0–1, indicating a good nutritional status; 43.2% of the patients had a score of 2–3, indicating a mild malnutrition or suspected malnutrition; 23.6% of the patients had a score of 4–8, indicating moderate malnutrition and requiring nutritional intervention and treatment; and 4.8% of the patients had a score ≥ 9 points, indicating severe malnutrition and an urgent need for symptomatic treatment and adequate nutritional support. In addition, the mean TP concentration was 67.08 ± 5.75 g/L, the serum ALB concentration was 41.35 ± 4.18 g/L, the HGB concentration was 131.07 ± 23.30 g/L, and the lymphocyte count was 1.80 ± 0.67 × 10^9^/L before surgery. Other patient demographic and clinical characteristics are shown in Table 1.

**Table 1:** Baseline characteristics of the patients who underwent GI surgery.

Characteristics	Total (*n* = 310)
Age (year)	61.74 ± 10.49
Sex	
Male	201 (64.8%)
Female	109 (35.2%)
BMI (kg/m^2^)	24.26 ± 3.08
Smoking habit	
No	190 (61.3%)
Yes	120 (38.7%)
Alcohol use	
No	220 (71.0%)
Yes	90 (29.0%)
Previous abdominal surgery	
No	253 (81.6%)
Yes	57 (18.4%)
Type of cancer	
GC	157 (50.6%)
CRC	153 (49.4%)
Tumor stage	
I-II	187 (60.3%)
III-IV	123 (39.7%)
Operation method	
Endoscopic surgery	196 (63.2%)
Open surgery	114 (36.8%)
Operation time (minute)	190.06 ± 64.50
PPOI	
No	275 (88.7%)
Yes	35 (11.3%)
PG-SGA (score)	
0–1	88 (28.4%)
2–3	134 (43.2%)
4–8	73 (23.6%)
≥ 9	15 (4.8%)
NRS2002 score ≥ 3	
No	142 (45.8%)
Yes	168 (54.2%)
Total protein level (g/L)	67.08 ± 5.75
Albumin level (g/L)	41.35 ± 4.18
Prealbumin level (mg/L)	253.00 ± 62.46
Triglyceride level (mmol/L)	1.25 ± 0.59
Total cholesterol level (mmol/L)	4.99 ± 0.96
Cholinesterase level (U/L)	8140.40 ± 1737.48
Uric Acid level (umol/L)	308.18 ± 77.86
K^+^ level (mmol/L)	4.16 ± 0.38
Mg^+^ level (mmol/L)	0.89 ± 0.06
P^+^ level (mmol/L)	1.09 ± 0.15
Hemoglobin level (g/L)	131.07 ± 23.30
Lymphocyte count (×10^9^/L)	1.80 ± 0.67

### Risk factors for PPOI after GI surgery according to univariate and multivariate analyses

In the univariate analyses, previous abdominal surgery (*p* = 0.034), tumor stage (*p* = 0.001), operation time (*p* = 0.004), PG-SGA (*p* < 0.001), NRS2002 score ≥ 3 (*p* = 0.011), total protein level (*p* = 0.010), albumin level (*p* < 0.001), prealbumin level (*p* < 0.001), cholinesterase level (*p* = 0.001), hemoglobin level (*p* < 0.001) and lymphocyte count (*p* = 0.026) were associated with PPOI (Table 2). Multivariate logistic regression indicated that previous abdominal surgery (OR = 3.186, 95%CI = 1.212–8.380, *p* = 0.019), tumor stage (OR = 2.847, 95%CI = 1.196–6.782, *p* = 0.018), operation time (OR = 1.006, 95%CI = 1.000–1.012, *p* = 0.036), PG-SGA score (OR = 2.551, 95% CI = 1.207–5.390, *p* = 0.014), albumin level (OR = 0.830, 95%CI = 0.691–0.997, *p* = 0.047), and hemoglobin level (OR = 0.978, 95%CI = 0.958–0.999, *p* = 0.040) were independent risk factors for PPOI (Table 3).

**Table 2:** Comparison of background data, tumor stage, operative duration, nutritional risk and nutritional parameters between the two groups.

	Without PPOI (*n* = 275)	With PPOI (*n* = 35)	*p* value
Age (year)	61.44 ± 10.46	64.14 ± 10.62	0.162
Sex			0.794
Male	179 (65.1%)	22 (62.9%)	
Female	96 (34.9%)	13 (37.1%)	
BMI (kg/m^2^)	24.32 ± 3.11	23.82 ± 2.78	0.33
Smoking habit			0.348
No	166 (60.4%)	24 (68.6%)	
Yes	109 (39.6%)	11 (31.4%)	
Alcohol use			0.949
No	195 (70.9%)	25 (71.4%)	
Yes	80 (29.1%)	10 (28.6%)	
Previous abdominal surgery			0.034
No	229 (83.3%)	24 (68.6%)	
Yes	46 (16.7%)	11 (31.4%)	
Type of cancer			0.328
GC	142 (51.6%)	15 (42.9%)	
CRC	133 (48.4%)	20 (57.1%)	
Tumor stage			
I-II	175 (63.6%)	12 (34.3%)	0.001
III-IV	100 (36.4%)	23 (65.7%)	
Operation method			
Endoscopic surgery	179 (65.1%)	17 (48.6%)	0.056
Open surgery	96 (34.9%)	18 (51.4%)	
Operation time (minute)	186.58 ± 64.81	217.57 ± 55.55	0.004
PG-SGA (score)			<0.001
0–1	83 (30.2%)	5 (14.3%)	
2–3	126 (45.8%)	8 (22.8%)	
4–8	61 (22.2%)	12 (34.3%)	
≥ 9	5 (1.8%)	10 (28.6%)	
NRS2002 score ≥ 3			0.011
No	133 (48.4%)	9 (25.7%)	
Yes	142 (51.6%)	26 (74.3%)	
Total protein level (g/L)	67.44 ± 5.54	64.26 ± 6.69	0.01
Albumin level (g/L)	41.82 ± 3.68	37.62 ± 5.83	<0.001
Prealbumin level (mg/L)	259.14 ± 57.61	204.68 ± 77.42	<0.001
Triglyceride level (mmol/L)	1.26 ± 0.55	1.18 ± 0.89	0.601
Total cholesterol level (mmol/L)	5.02 ± 0.96	4.73 ± 0.98	0.1
Cholinesterase level (U/L)	8276.51 ± 1673.54	7070.97 ± 1880.98	0.001
Uric Acid level (umol/L)	309.81 ± 77.40	295.35 ± 81.40	0.325
K^+^ level (mmol/L)	4.15 ± 0.39	4.21 ± 0.32	0.359
Mg^+^ level (mmol/L)	0.89 ± 0.06	0.89 ± 0.09	0.799
P^+^ level (mmol/L)	1.10 ± 0.14	1.04 ± 0.21	0.118
Hemoglobin level (g/L)	133.83 ± 20.48	109.40 ± 31.80	<0.001
Lymphocyte count (×10^9^/L)	1.82 ± 0.67	1.56 ± 0.63	0.026

**Table 3:** Independent risk factors for PPOI.

	OR (95% CI)	*p* value
Previous abdominal surgery	3.186 (1.212–8.380)	0.019
Tumor stage	2.847 (1.196–6.782)	0.018
Operation time	1.006 (1.000–1.012)	0.036
PG-SGA score	2.551 (1.207–5.390)	0.014
NRS2002 score ≥ 3	0.401 (0.109–1.476)	0.169
Total protein level	1.060 (0.951–1.181)	0.293
Albumin level	0.830 (0.691–0.997)	0.047
Prealbumin level	0.998 (0.988–1.008)	0.685
Cholinesterase level	1.000 (1.000–1.000)	0.7
Hemoglobin level	0.978 (0.958–0.999)	0.04
Lymphocyte count	1.073 (0.544–2.117)	0.838

### Establishment and validation of the diagnostic nomogram model

**Figure 2:**
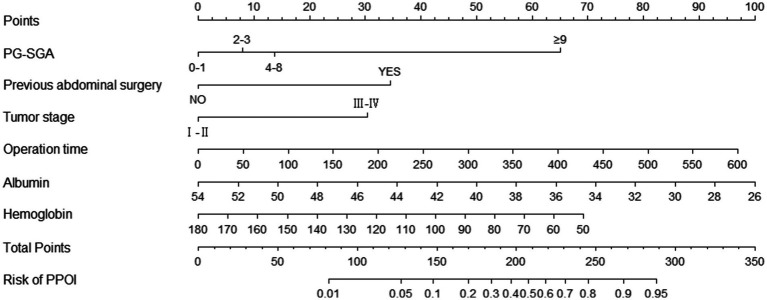
Nomogram for predicting PPOI.

**Figure 3:**
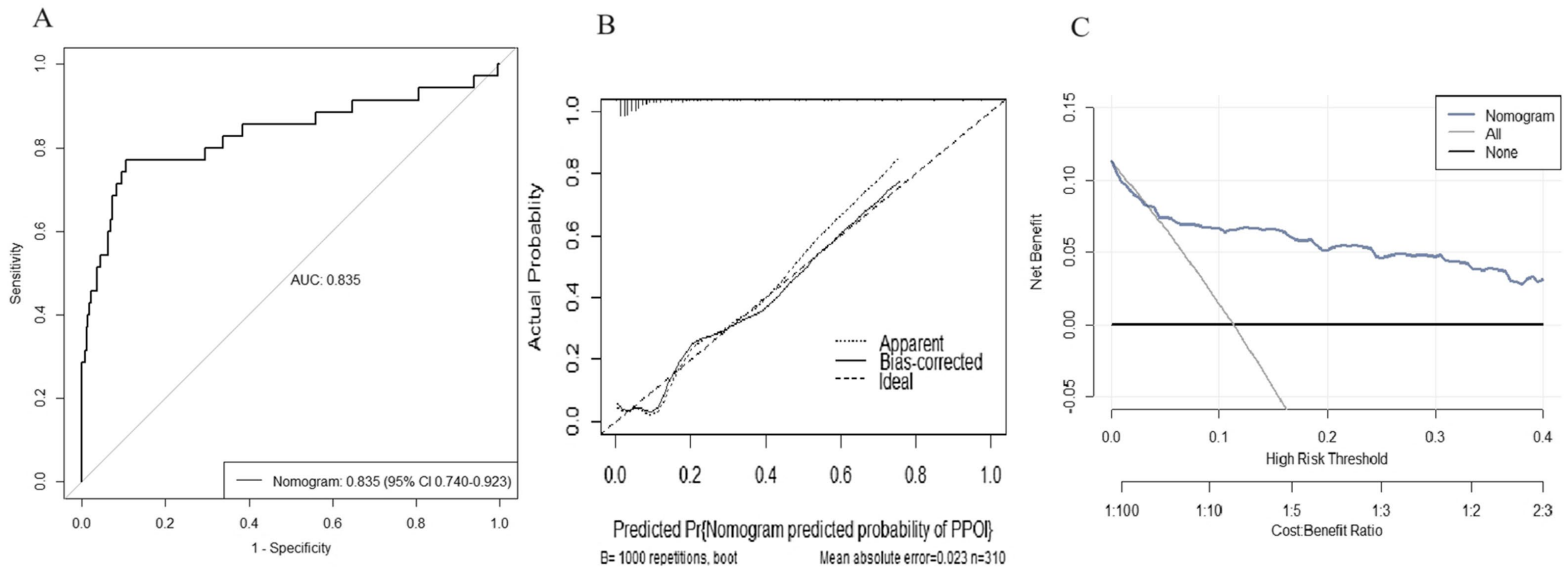
Performance of the nomogram model for predicting PPOI. **(A)** ROC curve analysis for the prediction of PPOI; **(B)** Calibration curve of the nomogram for predicting PPOI; **(C)** Decision curve analysis for predicting PPOI.

We used the independent predictors obtained via the multivariable logistic regression to construct a nomogram to predict PPOI (Figure 2). The ROC analysis revealed that the AUC value of the nomogram was 0.835, indicating that this model has excellent discriminative ability (Figure 3A). According to the calibration curve, the observed results were highly consistent with the predicted results (Figure 3B). In addition, the DCA revealed that the nomogram model is effective in clinical practice (Figure 3C).

### Clinical value of the nomogram compared with that of albumin level and PG-SGA score

**Figure 4:**
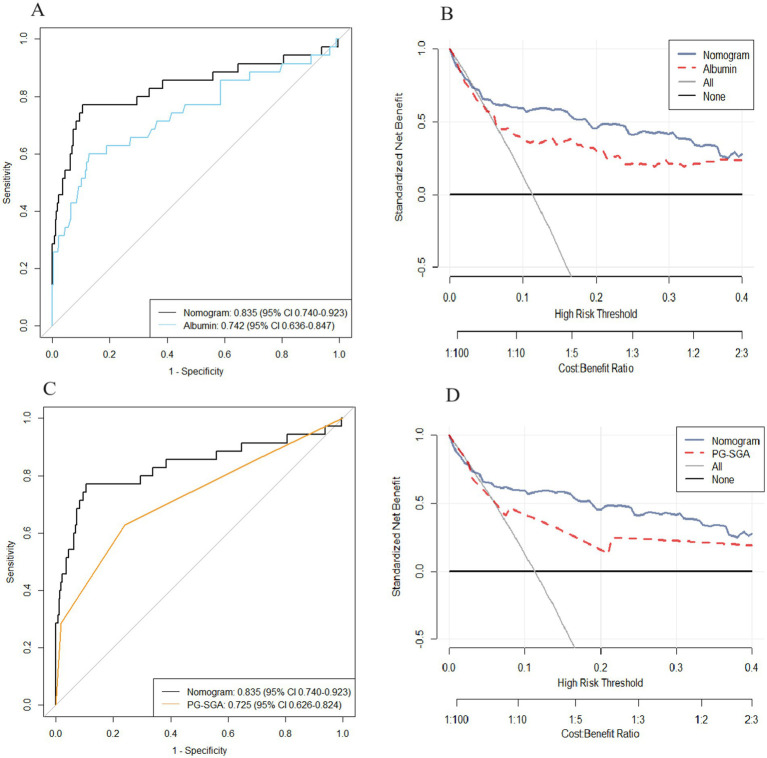
The area under the receiver operating characteristic curve and DCA curve of the nomogram were compared with those of albumin level **(A,B)** and PG-SGA score **(C,D)**.

We plotted ROC and DCA curves for the albumin level and the PG-SGA score, and the nomogram had better predictive power than the albumin level and the PG-SGA score in both the ROC curves and the DCA curves. (Figures 4A–D).

## Discussion

Nutritional factors were not considered in the development of prior predictive models for PPOI; thus, in this study, we conducted a comprehensive analysis of the variables associated with patient nutritional status. Independent predictors, including PG-SGA score, albumin level, hemoglobin level, previous abdominal surgery, operation time and advanced tumor stage (III-IV), were identified. These six variables were used to construct a novel nomogram for predicting PPOI. The nomogram was validated and exhibited strong discriminatory and calibration capabilities. Liang et al. found that age, postoperative opioid analgesics, postoperative K+, surgical method, and tumor stage were independent risk factors for PPOI, and used these variables to construct a nomogram with an area under the curve (AUC) of 0.836 (18). Guo et al. used the predictors of age, hypoproteinemia, high surgical difficulty, and postoperative use of opioid analgesics to construct a nomogram for predicting PPOI after rectal cancer surgery (19). ROC analysis showed that the AUC of the nomogram was 0.738.Similarly, albumin level and tumor stage were also predictors of PPOI in our study. In addition, history of previous abdominal surgery and duration of surgery were found to be predictors. Unlike other nomograms that included surgery-related predictors, our nomogram included preoperative nutrition-related predictors such as PG-SGA score and hemoglobin level, and the diagnostic performance (AUC = 0.835) of our nomogram was not inferior to other nomograms. Consequently, clinicians can combine this tool with the nutritional status of patients to assess the risk of PPOI more accurately and carry out corresponding nutritional intervention to prevent the occurrence of PPOI.

The incidence of PPOI in our study was 11.3%, which is similar to that reported in previous studies. A meta-analysis by Quiroga-Centeno et al. revealed that the incidence of PPOI after colorectal surgery was 9.56% (20). In an observational study of 2,400 consecutive patients, Chapuis et al. reported a PPOI incidence of 14.0% (21). However, in a related study by Mao et al., 88 of the 325 patients who underwent elective colorectal surgery (27%) experienced PPOI (22). The incidence of PPOI varies across previous studies, depending on the definition of PPOI. The prolonged duration of intestinal obstruction is an important factor in defining PPOI, and the duration of intestinal obstruction varies from 3 to 7 days in different studies (18). A generally accepted definition of PPOI should be established for future studies.

The PG-SGA, modified by Ottery and based on the SGA, is a nutritional screening tool specifically developed for patients with malignant tumors (23). According to the American Dietetic Association, the PG-SGA is recommended as the standard for nutritional evaluation in patients with malignant tumors (24). Unlike other nutritional assessment tools, the best feature of the PG-SGA is its inclusion of symptoms related to food intake and nutritional evaluation. This characteristic makes it more suitable for patients with GC or CRC cancer, who often experience various diet-related symptoms (25). A retrospective study revealed that the PG-SGA was more appropriate than the NRS2002 for evaluating the preoperative nutritional status of GC patients and had greater diagnostic efficiency (26). Zhang et al. reported that a high risk of malnutrition, as assessed by the PG-SGA, was associated with an increased risk of postoperative complications (27). Our current research involving patients undergoing radical resection of GC or CRC revealed that a high preoperative PG-SGA score is associated with an increased risk of PPOI. Therefore, for patients with severe malnutrition assessed by PG-SGA, improving the degree of malnutrition before surgery can prevent the occurrence of PPOI.

The clinical indicators commonly associated with malnutrition in cancer patients include albumin and hemoglobin levels, and albumin can serve as a sensitive marker for evaluating nutritional status (28). Patients with GI cancer often experience hypoalbuminemia due to factors such as GI obstruction, malabsorption, inhibition of liver synthesis by the tumor, and increased consumption of albumin caused by fast tumor cell proliferation and active cell metabolism (29). Hypoalbuminemia decreases colloid osmotic pressure and causes an accumulation of extracellular fluid between tissues, causing intestinal mucosal oedema, which therefore interferes with the normal exchange of ions, affects intestinal function and may be related to the occurrence of PPOI (30). Hardt et al. identified hypoalbuminemia as an independent risk factor for postoperative complications (31). Similarly, Baik et al.’s retrospective study revealed that the occurrence of postoperative complications was associated with hypoproteinaemia (32). Furthermore, patients with GI tumors frequently develop anemia resulting from GI bleeding, iron or folate deficiency, and inflammation (33). Anemia is a well-known predictor of postoperative complications (34). In this study, we specifically investigated the relationship between preoperative albumin and hemoglobin levels and the occurrence of PPOI, confirming that both albumin and hemoglobin levels are predictive factors for PPOI. Preoperative improvement of anemia and hypoproteinemia can prevent the occurrence of PPOI.

A prolonged operation time increases the extent of surgical invasion, leading to tissue damage and inflammation at the surgical site due to intraoperative nociception, resulting in the production of a significant amount of inflammatory mediators (35). Inflammatory cytokines can promote the infiltration of white blood cells into smooth muscle and nerve tissues, causing inflammation-induced damage (36). They also inhibit smooth muscle calcium ion channels and disrupt the balance between the sympathetic and parasympathetic nerves, thereby affecting the lower GI smooth muscle contraction force (37). This study revealed a significantly increased rate of PPOI in patients whose surgery was prolonged. Similar conclusions have been drawn in previous studies, identifying surgery duration as an independent risk factor for PPOI (38). Bai et al. emphasized that tumor stage is an important predictor of PPOI (39). Consistent with this finding, our study revealed that patients with advanced tumor stages were more prone to developing PPOI. These patients often exhibit a poor nutritional status, have larger surgical wounds, and underwent a difficult and prolonged surgery, all of which are factors contributing to the development of PPOI (40). Previous abdominal surgery may lead to changes in the anatomy of related organs, resulting in visceral adhesions, thus increasing the complexity of the operation, prolonging the operation and therefore the risk of complications (41). Our study revealed that patients with a previous history of abdominal surgery were prone to PPOI. However, Lin et al. reported that there was no significant difference in the incidence of PPOI between patients with and without a previous history of abdominal surgery (42), which contrasts with the results of our study. More studies are needed to further confirm whether previous abdominal surgery can affect the occurrence of PPOI.

The nomogram integrates multiple factors (including demographic and clinical pathological characteristics) into a quantitative model and has been proven to have excellent predictive performance and benefits for making clinical decisions. We compared the nomogram involving more variables with albumin level and PG-SGA score. The results indicated that this nomogram has better predictive ability than albumin level or PG-SGA score. Furthermore, the DCA confirmed that our nomogram has better clinical utility than albumin level or PG-SGA score.

This study has certain advantages. First, in most previous studies, researchers focused on investigating the influence of perioperative-related factors on the occurrence of PPOI, and, in this study, we provide nutrition-related evidence for predicting the occurrence of PPOI. Second, the ROC curve and calibration of the prediction model were both reliable in the internal validation. Furthermore, the results of the decision curve analysis confirmed that the predictive model was clinically useful. However, this study has several limitations. First, this was a single-center retrospective study, which may have certain bias. Second, robust external validation of the nomogram was not performed. Therefore, these results need to be further validated in subsequent studies.

## Conclusion

This retrospective cohort study revealed that PG-SGA score, previous abdominal surgery, operation time, advanced tumor stage, and albumin and hemoglobin levels were independent predictors of PPOI. The nomogram based on these predictors has good predictive accuracy and clinical practicability.

## Data Availability

The original contributions presented in the study are included in the article/Supplementary material, further inquiries can be directed to the corresponding authors.
